# Soil bacterial and fungal communities of six bahiagrass cultivars

**DOI:** 10.7717/peerj.7014

**Published:** 2019-05-29

**Authors:** Lukas Beule, Ko-Hsuan Chen, Chih-Ming Hsu, Cheryl Mackowiak, Jose C.B. Dubeux Jr., Ann Blount, Hui-Ling Liao

**Affiliations:** 1 Molecular Phytopathology and Mycotoxin Research, Georg-August Universität Göttingen, Goettingen, Germany; 2 North Florida Research and Education Center, University of Florida, Quincy, FL, United States of America; 3 North Florida Research and Education Center, University of Florida, Marianna, FL, United States of America

**Keywords:** Bahiagrass (*Paspalum notatum* Flüggé), Cultivars, Soil bacterial community, Soil fungal community

## Abstract

**Background:**

Cultivars of bahiagrass (*Paspalum notatum* Flüggé) are widely used for pasture in the Southeastern USA. Soil microbial communities are unexplored in bahiagrass and they may be cultivar-dependent, as previously proven for other grass species. Understanding the influence of cultivar selection on soil microbial communities is crucial as microbiome taxa have repeatedly been shown to be directly linked to plant performance.

**Objectives:**

This study aimed to determine whether different bahiagrass cultivars interactively influence soil bacterial and fungal communities.

**Methods:**

Six bahiagrass cultivars (‘Argentine’, ‘Pensacola’, ‘Sand Mountain’, ‘Tifton 9’, ‘TifQuik’, and ‘UF-Riata’) were grown in a randomized complete block design with four replicate plots of 4.6 × 1.8 m per cultivar in a Rhodic Kandiudults soil in Northwest Florida, USA. Three soil subsamples per replicate plot were randomly collected. Soil DNA was extracted and bacterial 16S ribosomal RNA and fungal ribosomal internal transcribed spacer 1 genes were amplified and sequenced with one Illumina Miseq Nano.

**Results:**

The soil bacterial and fungal community across bahiagrass cultivars showed similarities with communities recovered from other grassland ecosystems. Few differences in community composition and diversity of soil bacteria among cultivars were detected; none were detected for soil fungi. The relative abundance of sequences assigned to nitrite-oxidizing *Nitrospira* was greater under ‘Sand Mountain’ than ‘UF-Riata’. Indicator species analysis revealed that several bacterial and fungal indicators associated with either a single cultivar or a combination of cultivars are likely to be plant pathogens or antagonists.

**Conclusions:**

Our results suggest a low impact of plant cultivar choice on the soil bacterial community composition, whereas the soil fungal community was unaffected. Shifts in the relative abundance of *Nitrospira* members in response to cultivar choice may have implications for soil N dynamics. The cultivars associated with presumptive plant pathogens or antagonists indicates that the ability of bahiagrass to control plant pathogens may be cultivar-dependent, however, physiological studies on plant-microbe interactions are required to confirm this presumption. We therefore suggest that future studies should explore the potential of different bahiagrass cultivars on plant pathogen control, particularly in sod-based crop rotation.

## Introduction

Bahiagrass (*Paspalum notatum* Flüggé), native to South America (Burton, 1967), is a widespread, warm-season perennial, commonly used as pasture in the Southeastern USA. Following its introduction into many countries worldwide, the sod-forming grass is also common in Australia and Japan (Hirata, 2000; Wilson, 1987) and has become naturalized in the USA. It was first introduced into the USA in 1913 (Scott, 1920) and is extensively cultivated on more than 1.5 million hectares in southeast USA, making it the most common and widely used perennial grass across southern states (Newman, Vendramini & Blount, 2011). Bahiagrass grows well in sandy, low fertile soils, requires low inputs, and it exhibits tolerance towards short-term drought and flooding events as well as continuous cattle stocking (Gates, Quarin & Pedreira, 2004; Newman, Vendramini & Blount, 2011). Low winter temperatures and aridity limit its geographic distribution (Gates, Quarin & Pedreira, 2004). ‘Pensacola’, ‘Tifton 9’, ‘TifQuik’, and ‘UF-Riata’ are among the most popular cultivars in the Southeastern USA. They exhibit differences in growth habit, cold tolerance, seasonal and total yield, seed production and grazing tolerance (Newman, Vendramini & Blount, 2011). Cultivars also can differ in their resistance to diseases (Hancock et al., 2010; Trenholm, Cisar & Unruh, 2011). Further, cultivar-specific nutrient use efficiencies may reduce nitrate leaching and fertilizer input costs (Wiesler & Horst, 1993; Liu, Hull & Duff, 1997; Baligar, Fageria & He, 2001). Therefore, cultivar choice is an important factor for the maintenance of soil health.

It is well established that plant community composition and diversity influences the belowground microbial community and vice-versa (Berg, 2009; Berg & Smalla, 2009; Van der Heijden et al., 1998; Kourtev, Ehrenfeld & Häggblom, 2003; Kowalchuk et al., 2002; Lange et al., 2015; Reynolds et al., 2003; Wardle et al., 2004; Zak et al., 2003). Beneficial plant-microbe interactions, such as mycorrhizal symbiosis or root colonization of plant growth-promoting rhizobacteria (PGPR) are known to enhance host plant growth (Artursson, Finlay & Jansson, 2006; Lugtenberg & Kamilova, 2009), pathogen resistance (Azcón-Aguilar & Barea, 1997; Harrier & Watson, 2004; Van Loon, Bakker & Pieterse, 1998; Maherali & Klironomos, 2007), and abiotic stress tolerance (Evelin, Kapoor & Giri, 2009; Vurukonda et al., 2016; Wu, Zou & Xia, 2006; Yang, Kloepper & Ryu, 2009). Whereby belowground, mycorrhiza symbionts depend on organic carbon supply via host roots (Smith & Read, 2008) and PGPR can be attracted via root exudates (Badri & Jorge, 2009; Somers, Vanderleyden & Srinivasan, 2004), creating a complex plant-microbe-soil feedback system (Miki et al., 2010). Emerging evidence shows that plant cultivars can be one of the factors affecting the composition of the rhizosphere microbiome (Briones et al., 2002; Dalmastri et al., 1999; Diab El Arab, Vilich & Sikora, 2001; Germida & Siciliano, 2001; Schweitzer et al., 2008). Different grass species have been shown to be capable of altering soil microbial communities, mainly due to differences in nutrient acquisition strategies and rhizodeposits (Bardgett et al., 1999; Grayston et al., 1998; Vandenkoornhuyse et al., 2003). A few studies reported that rhizosphere bacterial populations vary across different grass cultivars (Miller, Henken & Veen, 1989; Rodrigues et al., 2016), whereas the potential effect of different grass cultivars on the composition of fungal communities remains widely unexplored. Identifying alterations of the soil microbiome by cultivar choice is of importance as specific microorganisms can have specific lifestyles, including mutualism, parasitism or involvement in diverse saprotrophic activities. These processes are directly linked to the fitness of the host plants and soil fertility.

Alterations of belowground microbial communities can have significant impact on plant performance. In several managed grassland ecosystems, Proteobacteria, Acidobacteria, Actinobacteria, and Bacteroidetes have been found to be the most abundant soil bacterial phyla (Cao et al., 2017; Kaiser et al., 2016; Nacke et al., 2011; Rodrigues et al., 2016; Zhou et al., 2003). Members of these phyla contribute to essential soil functions, such as biological nitrogen fixation (BNF) (Baldani et al., 1997). Further, beneficial rhizobacteria can stimulate plant growth via the production of plant hormones, suppress soil-borne plant pathogens, supply nutrients to plants and improve soil structure (Berg, 2009; Hayat et al., 2010; Van der Heijden, Bardgett & Van Straalen, 2008; Weller et al., 2002). Hence, PGPR such as *Arthrobacter*, *Azotobacter*, *Burkholderia*, and *Pseudomonas* species have been used to enhance agricultural production for decades (Bhattacharyya & Jha, 2012; Vessey, 2003). Besides bacteria, symbiotic associations with mycorrhizal fungi can improve plant resistance to pathogens (Selosse, Baudoin, & Vandenkoornhuyse, 2004; Wehner et al., 2010) as well as improve plant nutrition, particularly by enhancing plant phosphorus (P) acquisition (Li et al., 2006; Smith, Mette Grønlund & Andrew Smith, 2011; Smith, Smith & Jakobsen, 2003). Many arbuscular mycorrhizal (AM) fungi communities under grass have been shown to be dominated by the families Glomeraceae, Gigasporaceae and Acaulosporaceae (Hiiesalu et al., 2014; Oehl et al., 2005; Xu et al., 2017). In some grassland soils, the genus *Glomus* was identified as the most abundant AM fungi (Gai et al., 2009; Wang et al., 2003). *Glomus* is the largest genus of AM fungi described (Schwarzott, Walker & Schüssler, 2001). In association with peanut (*Arachis hypogaea*) and lettuce (*Lactuca sativa*) plants, *Glomus* spp. were demonstrated to promote plant growth, P and micronutrient uptake (Krishna & Bagyaraj, 1984) and increased drought tolerance (Ruiz-Lozano, Azcon & Gomez, 1995).

Using next generation amplicon sequencing, the aim of this study was to determine whether different bahiagrass cultivars interactively influence the belowground microbial community composition and diversity. To achieve this aim, we recovered bacterial 16S ribosomal RNA (16S rRNA) and fungal ribosomal internal transcribed spacer (ITS) 1 gene sequences from soil samples of six different bahiagrass cultivars grown in a randomized complete-block design. We hypothesized that bahiagrass cultivar choice affects the microbial community composition and diversity of both, soil bacteria and fungi. Given the significant role of soil microorganisms in soil nutrient cycling and plant nutrition, our research outcomes can provide insight into bahiagrass-associated soil bacterial and fungal communities, as well as the plant-microbe-soil feedback system among grass cultivars and better our understanding of the grassland ecosystem.

## Material and Methods

### Study site

The experimental site (30.8733 N, 85.1894 W, 33 m above sea level) is located in Northwest Florida (Jackson County), USA. The soil was characterized as a fine-loamy, kaolinitic, thermic Rhodic Kandiudults of the Orangeburg series (National Cooperative Soil Survey U.S.A., 2019). In June 2005, six different bahiagrass cultivars (‘Argentine’, ‘Pensacola’, ‘Sand Mountain’, ‘Tifton 9’, ‘TifQuik’, and ‘UF-Riata’) were established, in a randomized complete-block design, with four replicate plots of 4.6 × 1.8 m per cultivar. All plots were treated the same for harvesting procedures and fertilization rates. Bahiagrass cultivars were harvested five times to a 5-cm stubble height during the growing season (May to October), which was conducted at five-weeks intervals. The plots were grown under low-fertilizer inputs and received no nitrogen (N) fertilization for the duration of this study. From May to August 2015, the plots received 7.3 kg P ha^−1^, 197.1 kg K ha^−1^, 67.3 kg Mg ha^−1^, and 141.2 kg S ha^−1^. From April to August 2016, the plots received 29.4 kg P ha^−1^, 239.9 kg K ha^−1^, 33.6 kg Mg ha^−1^, and 70.6 kg S ha^−1^.

Soil characteristics were assessed prior to the planting in 2005. Five soil cores (Ø: 2.54 cm) of 0–15 cm depth were taken within each replicate plot to receive a total of 30 soil subsamples per block. One composite soil sample form each of the four blocks was analysed for soil pH (1:2, soil:water), Mehlich-1 extractable nutrients and calculated cation exchange capacity were determined by a commercial lab (Waters Agricultural Laboratories, Inc., Camilla, GA, USA). Soil properties are reported in Table S1.

### Soil sampling and soil DNA extraction

Three randomly selected soil samples per replicate plot, resulting in twelve soil samples per cultivar, were taken in late April 2017 (mean temperature in April 2017: 22 °C [6–33 °C], sum of precipitation in April 2017: 51.8 mm [0.0–25.9 mm day^−1^]). Soil cores (Ø: 2 cm) of 10 cm depth were stored at 4 °C during transportation to the laboratory (one hour). Upon arrival in the laboratory, soil samples were homogenized and sieved at ≤ 2 mm. Aliquots of each soil sample were transferred to 2 ml Eppendorf tubes, frozen in liquid N_2_ for 3 min and subsequently stored at −80 °C until DNA extraction. Total soil DNA was extracted using Qiagen’s DNeasy® PowerSoil® Kit (Qiagen Inc., CA, USA) following the manufacturer’s instructions. Quality and quantity of the extracts were assessed using a spectrophotometer (NanoDrop (ND-ONE-W), ThermoFisher Scientific, Waltham, MA, USA).

### Amplicon sequencing

To assess community compositions of soil bacteria and fungi, three-step PCRs targeting the bacterial V4 region of the 16S rRNA and fungal ITS1 genes were modified according to Chen et al. (2018). Briefly, bacterial 16S rRNA and fungal ITS1 genes were amplified for 10 PCR cycles (first-step PCR) using primer pair 515F (5′- GTGCCAGCMGCCGCGGTAA-3′)/806R (5′-GACTACHVGGGTWTCTAAT-3′) and ITS1F (5′-CTTGGTCATTTAGAGGAAGTAA-3′)/ITS2 (5′-GCTGCGTTCTTCATCGAT GC-3′), respectively. Another 10 PCR cycles (2nd-step PCR) were used to add six frameshifting primers as well as the sequencing primer. The frameshifting primers consisted of the respective primer pair used in the first-step PCR with frameshifting nucleotides to create sequence diversity in order to overcome the sequence bias within the initial bases and, thus, increase data yield (Lundberg et al., 2013). Finally, error tolerant barcodes were added running additional 10 PCR cycles (3rd-step PCR). Prior to pooling, 3rd-step PCR products were individually purified using bead-cleanup (AMPure XP, Beckman Instruments, Brea, CA, USA). Quality and quantity of the PCR products were assessed using a spectrophotometer (NanoDrop™). In addition, PCR products were screened on 1.7% (w/v) agarose gels to verify product size and quantity. The 144 barcoded PCR products were pooled and sequenced with one Illumina (Illumina Inc., San Diego, CA, USA) Miseq Nano (v2 250 bp, 500 Mb sequencing capacity) at Duke Center for Genomic and Computational Biology (GCB, Durham, NC, USA). Amplicon sequencing data have been deposited at NCBI Short Read Archive (SRP143584).

### Amplicon sequencing data analysis

Sequence quality of obtained demultiplexed forward and reverse sequences was assessed by FastQC (Andrews, 2010). Forward primers were removed using cutadapt version 1.15 (Martin, 2011). Reverse sequences were not used due to low quality and merging with forward reads (Nguyen et al., 2015). The datasets were imported in QIIME2 verison 2018.11. Data were quality-filtered and chimeric sequences were removed employing DADA2 (Callahan et al., 2016). Forward reads truncated to 200 bp were processed for both bacterial and fungal datasets. The obtained 189,521 bacterial and 138,263 fungal quality-filtered reads were de novo assembled at 97% genetic identity using VSEARCH (Rognes et al., 2016). For taxonomic assignment, sequences were aligned to the Silva SSURef 132 NR (Quast et al., 2013) and UNITE version 7.2 database (Kõljalg et al., 2013) using BLAST+ (Camacho et al., 2009) in QIIME2 for 16S and ITS1, respectively. Singletons and non-bacterial and non-fungal reads were removed from the obtained operational taxonomic unit (OTU) tables. The OTU tables were rarefied to 1,200 for bacterial 16S rRNA and 600 randomly selected reads per sample for fungal ITS1 in QIIME2 (rarefaction curves are presented in Fig. S1).

### Statistical analyses

Shannon-Wiener and Simpson’s diversity indices, Chao1 richness estimate, and Simpson’s evenness of samples from rarefied OTU tables were calculated using the ‘diversity’-function in the R package ‘vegan’ version 2.4-5 (Oksanen, 2017). Non-metric multidimensional scaling (NMDS) based on Bray-Curtis dissimilarity matrices was conducted using the ‘metaMDS’-function in ‘vegan’. Significant differences in alpha diversity metrics (Shannon-Wiener and Simpson’s diversity indices, Chao1 richness estimate, and Simpson’s evenness) were tested using Kruskal-Wallis test with multiple comparison extension (‘kruskalmc’-function in the ‘pgirmess’ R package version 1.6.9 (Giraudoux et al., 2018). We further tested for significant differences in the relative abundance of taxonomic groups at all taxonomic levels (phylum to species) using one-way ANOVA with Tukey’s HSD test or Kruskal-Wallis test with multiple comparison extension as described above. Indicator species of individual plant cultivars as well as a combination of cultivars were identified using the ‘multipatt’-function using 999 permutations in the ‘indicspecies’ R package version 1.7.6 (De Caceres, 2013).

Differences in community composition among cultivars were tested using permutational multivariate analysis of variance (PERMANOVA) and complementary test for homogeneity of dispersions (PERMDISP) using 9,999 permutations employing the ‘beta-group-significance’-function in QIIME2 version 2018.11. Results for both PERMANOVA and PERMDISP were corrected for multiple comparison using Benjamini–Hochberg correction. Complementary, we performed Analysis of similarities (ANOSIM) using 9,999 permutations using the same function as for PERMANOVA and PERMDISP, yielding in similar results. Here, we report the results from PERMANOVA and PERMDISP. Test results with *p* < 0.05 were considered statistically significant. All statistical analyses were executed in R version 3.4.3 (R Core Team, 2017).

## Results

### Microbial community composition across six bahiagrass cultivars

**Figure 1:**
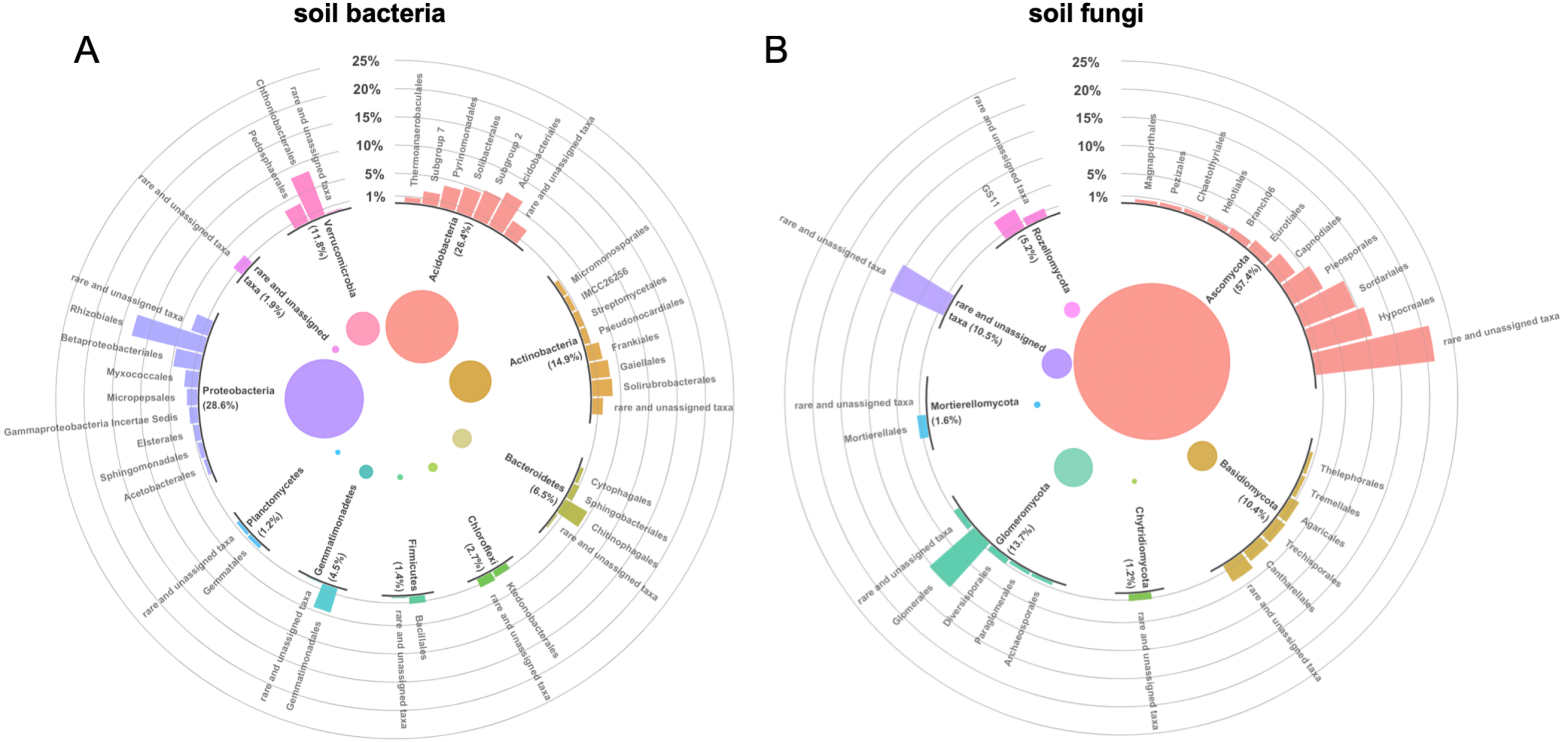
(A) Soil bacterial and (B) fungal community composition across plots of six different bahiagrass (*Paspalum notatum* Flüggé) cultivars (*n* = 72) in a Rhodic Kandiudults soil in Northwest Florida, USA. Rare (<0.5% relative abundance) phyla and orders were grouped with unassigned taxa.

The most abundant soil bacterial phyla were Proteobacteria (28.6 ± 8.5%), Acidobacteria (26.4 ± 10.2%), Actinobacteria (14.9 ± 3.3%), and Verrucomicrobia (11.8 ± 9.0%) (Fig. 1A). The Proteobacteria were divided into Alpha- (18.5 ± 8.1%), Delta-(3.0 ± 1.2%), and Gammaproteobacteria (7.1 ± 1.4%). Rhizobiales (12.9 ± 7.9%), Chthoniobacterales (8.2 ± 8.9%), Acidobacterales (6.7 ± 4.3%), and ‘Subgroup 2’ (5.3 ± 8.5%) were the dominant bacterial orders in soil (Fig. 1A). Sequences that matched closest with Candidatus *Udaeobacter* (8.2 ± 6.7%), *Bradyrhizobium* (4.7 ± 7.0%), and Candidatus *Solibacter* (2.7 ± 1.6%) were the most abundant in occurrence bacterial genera across cultivars.

Ascomycota (54.7 ± 13.8%), Glomeromycota (13.7 ± 7.5%), Basidiomycota (10.4 ± 8.6%), and Rozellomycota (9.8 ± 7.0%) were the most abundant fungal phyla (Fig. 1B). On class level, the fungal communities were dominated by Sordariomycetes (28.9 ± 14.0%), Glomeromycetes (11.7 ± 7.0%), Dothideomycetes (9.4 ± 9.2%), and Agaricomycetes (8.4 ± 8.6%). The most dominant fungal orders were Hypocreales (11.2 ± 9.0%), Sordariales (10.5 ± 7.0%), Glomerales (9.8 ± 10.2%), and Pleosporales (6.0 ± 5.0%) (Fig. 1B). Sequences that matched closest with *Penicillium* (1.9 ± 1.9%), *Fusarium* (1.8 ± 1.2%), and *Mortierella* (1.4 ± 1.7%) were the dominant fungal genera.

### Soil microbial diversity under different bahiagrass cultivars

**Figure 2:**
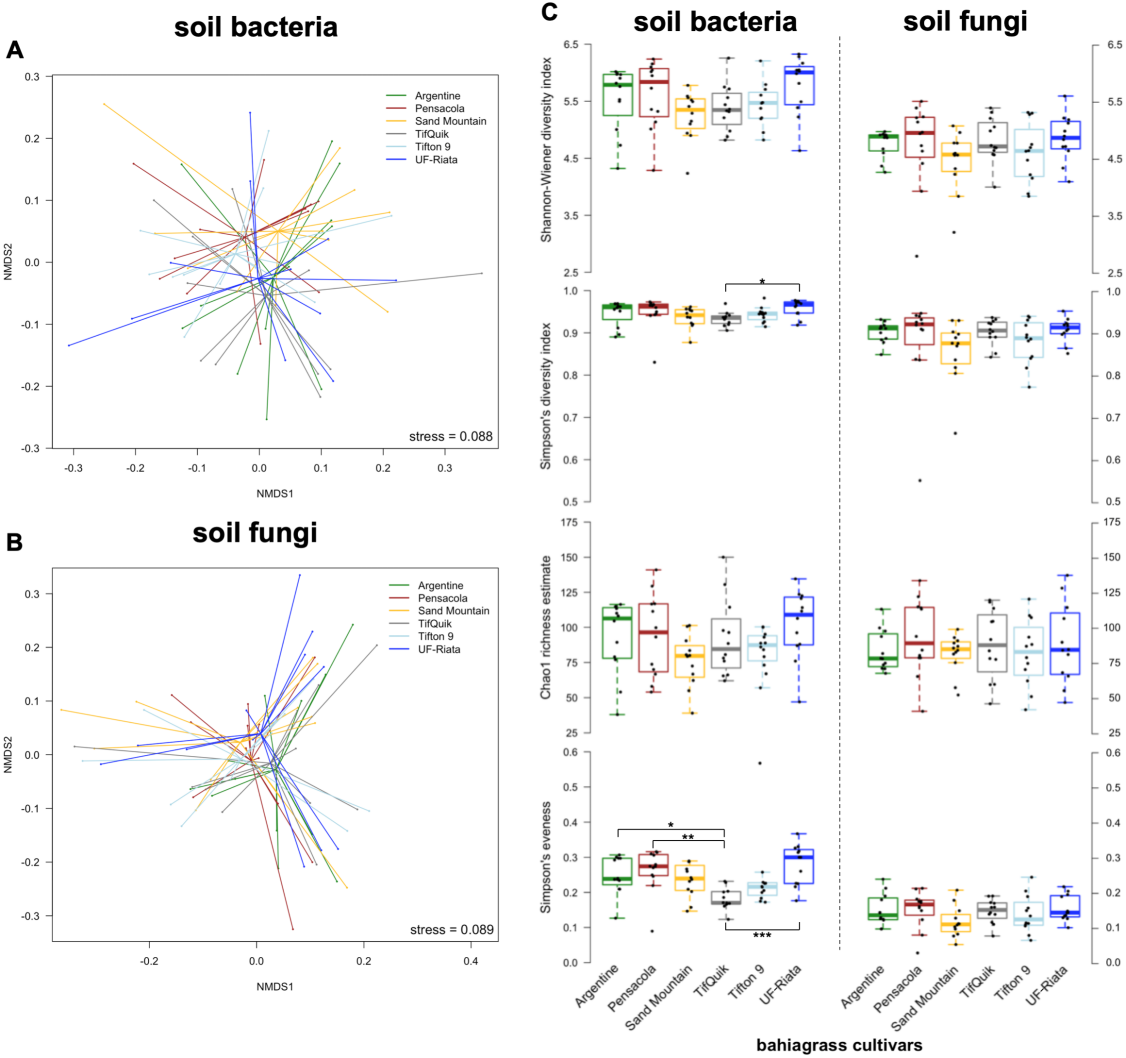
Microbial community composition and diversity in soil of six different bahiagrass (*Paspalum notatum* Flüggé) cultivars (*n* = 12 for each cultivar) in a Rhodic Kandiudults soil in Northwest Florida, USA. (A) Non-metric multidimensional scaling ordination (NMDS) of Bray-Curtis dissimilarity matrices of the soil bacterial and (B) fungal community, and (C) diversity, richness, and evenness metrics of soil bacterial and fungal communities. Black dots represent individual data points. Brackets indicate statistically significant differences among cultivars (Kruskal-Wallis test with multiple comparison extension at *p < 0.05, **p < 0.01, and ***p < 0.001).

To understand whether bahiagrass cultivar is among the factors shaping the community of soil microorganisms, the community composition and diversity of soil microorganisms across different cultivars was compared. Differences in the soil bacterial community composition between Argentine and Sand Mountain (*p* = 0.022) as well as Argentine and TifQuik were detected (*p* = 0.022) (Table S2). Soil fungal community composition did not differ among cultivars (Table S2), which is demonstrated by the clustering of the cultivars in the NMDS (Fig. 2B).

The different cultivars did not differ in their bacterial and fungal diversity based on the Shannon-Wiener diversity index; however, using Simpson’s diversity index, a greater diversity of bacteria in soil of UF-Riata compared to TifQuik was observed (*p* = 0.015) (Fig. 2C). No differences in bacterial and fungal richness were observed among cultivars, and Simpson’s evenness revealed lower bacterial species evenness in soil under TifQuik than Argentine (*p* = 0.023), Pensacola (*p* = 0.002), and UF-Riata (*p* < 0.001) (Fig. 2C).

### Shifts of relative abundance and indicator species in response to different bahiagrass cultivars

**Figure 3:**
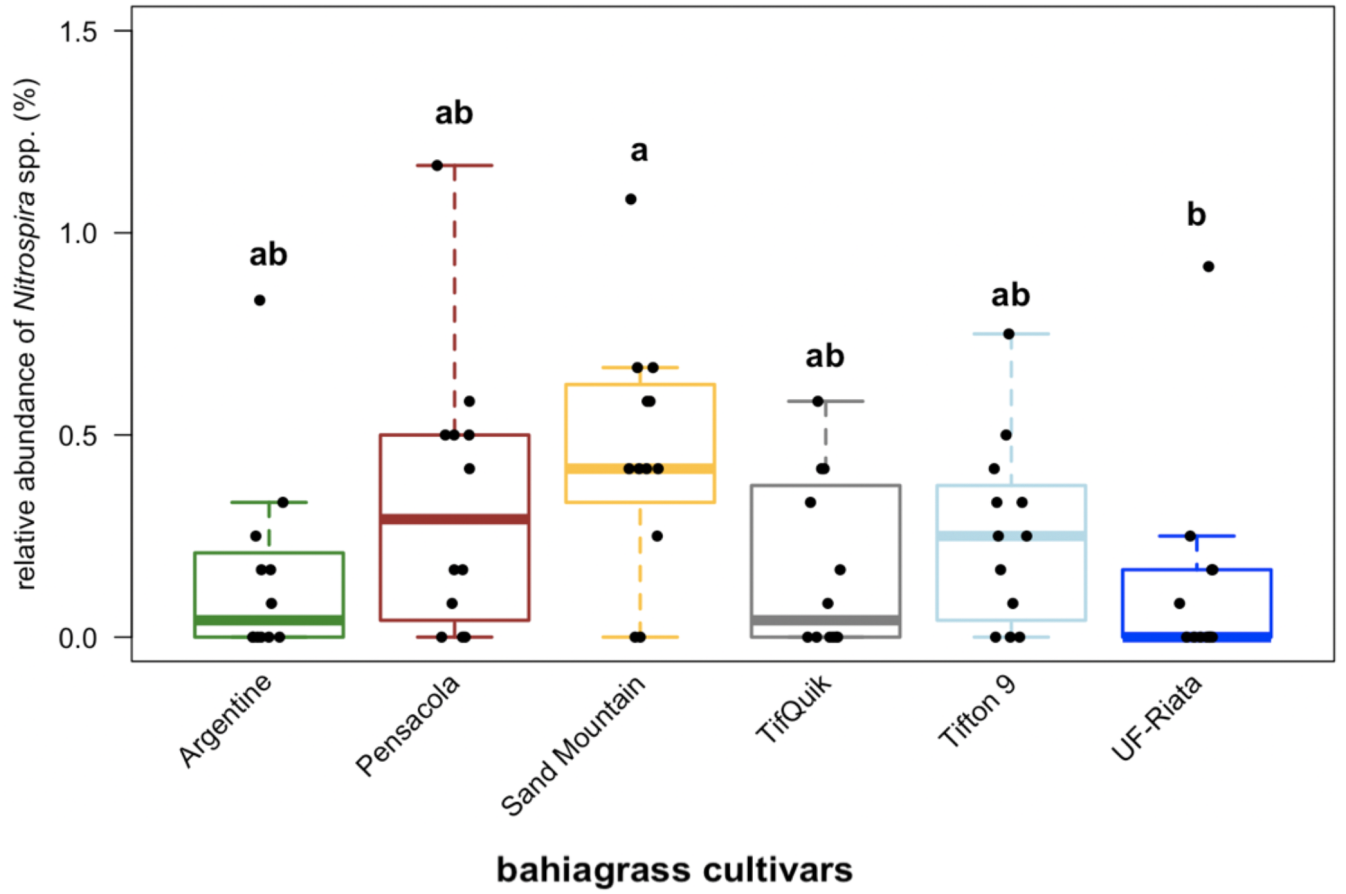
Relative abundance of *Nitrospira.* in soil of six different bahiagrass (*Paspalum notatum* Flüggé) cultivars (*n* = 12 for each cultivar) in a Rhodic Kandiudults soil in Northwest Florida, USA. Black dots represent individual data points. Different lowercase letters indicate statistically significant differences among cultivars (Kruskal-Wallis test with multiple comparison extension at *p* < 0.05).

We detected only one relative abundance shift of the bacterial and none of the fungal taxonomic groups among cultivars. The shift was found for the bacterial genus *Nitrospira*, where Sand Mountain was showing greater relative abundance than UF-Riata (*p* = 0.049) (Fig. 3A).

Out of 425 bacterial OTUs, there were 13 indicator species for individual cultivars as well as a combination of cultivars from which the majority of indicators (8 out of 13) were identified as Proteobacteria. Sand Mountain and TifQuik were the only individual cultivars that harboured distinct indicator species from the other cultivars in this study. The remaining indicators species were assigned to a combination of cultivars (Table S3). An OTU that matched closest to *Pajaroellobacter* (Deltaproteobacteria) and one that was assigned to *Bauldia* (Alphaproteobacteria) were associated with Sand Mountain (*p* ≤ 0.048) (Table S3). For the cultivar TifQuik, an OTU of the order ‘54-9’ (Anaerolineae) was identified as an indicator species (*p* = 0.019) (Table S3). Further, an OTU matched closest to *Haliangium* (Deltaproteobacteria) was characterised as an indicator species for Pensacola and Tifton 9 (*p* = 0.008) (Table S3). The presence of an unassigned member of the family Nitrosomonadaceae (Gammaproteobacteria) was identified as an indicator for all cultivars but Argentine (*p* = 0.017) (Table S3).

Of a total of 180 fungal OTUs, six indicator species were detected (Table S4). One OTU of the family Ceratobasidiaceae (Agaricomycetes) was characterized as an indicator for Pensacola, Sand Mountain, and Tifton 9 (*p* = 0.016) (Table S4). Sand Mountain, TifQuik, Tifton 9, and UF-Riata were characterized by a fungal OTU assigned to the family Orbiliaceae (*p* = 0.040) (Table S4).

## Discussion

### Soil bacterial communities across bahiagrass cultivars

The soil bacterial communities across managed bahiagrass cultivars exhibited parallels to the communities of diverse grassland ecosystems at phylum and class level. For example, the top three dominant soil bacterial phyla across all bahiagrass plots (Proteobacteria, Acidobacteria, and Actinobacteria) as well as the dominance of the Alpha-, Delta-, and Gammaproteobacteria were also reported for managed grassland soils (Cao et al., 2017; Nacke et al., 2011; Rodrigues et al., 2016; Zhou et al., 2003). Further, the greater relative abundance of the phyla Acidobacteria and Actinobacteria agrees with other studies investigating bacterial communities in grassland soils (Kaiser et al., 2016; Nacke et al., 2011; Rodrigues et al., 2016; Will et al., 2010).

The most abundant bacterial genus that was taxonomically assigned across cultivars of bahiagrass, Candidatus *Udaeobacter*, is ubiquitous in soils and frequently recovered using 16S rRNA gene sequencing approaches. Recently, Brewer et al. (2016) reported that an affiliate of this genus, Candidatus *Udaeobacter copiosus*, can account for almost one third of the soil bacterial taxa in grasslands. Further, Candidatus *Udaeobacter copiosus* has shown dominance in soil samples even across geographic distance (Brewer et al., 2016). Despite its great relative abundance in soils worldwide, the ecology and physiology of members of the genus Candidatus *Udaeobacter* largely remain unknown.

Our second most abundant soil bacterial genus (*Bradyrhizobium*) that matched our sequences was previously found as one of the most prominent genera in other grassland ecosystems (Brewer et al., 2016; McCaig, Glover & Prosser, 1999; Thomson et al., 2010). Many *Bradyrhizobium* species have the ability to denitrify (Bedmar, Robles & Delgado, 2005; Fernández et al., 2008; Kaneko et al., 2002; Mesa, Göttfert & Bedmar, 2001) and are proposed to play a key role in denitrification (Jones et al., 2016). Moreover, several *Bradyrhizobium* affiliates are capable of fixing atmospheric N_2_ and are considered to contribute significantly to BNF in soils (Zahran, 1999). The abundance of *Bradyrhizobium*, however, cannot serve as an indicator of their N_2_ fixation rates as shown in a recent study on native switchgrass (*Panicum virgatum*) (Bahulikar et al., 2014). Thus, although a genetic potential for denitrification and BNF is given by our second most dominant soil bacterial genus, its contribution to N cycling in soil of bahiagrass remains unclear and requires further investigations on functional level.

In line with the other dominant genera that we taxonomically assigned, the genus Candidatus *Solibacter*, our third most abundant genus, has been reported as one of the top genera recovered from grassland soils (Kaiser et al., 2016). Even for the most frequently investigated affiliate of the genus, Candidatus *Solibacter usitatus*, detailed ecological and physiological information is still lacking (Dedysh et al., 2017; Ward et al., 2009).

### Soil fungal communities across bahiagrass cultivars

In line with previous results from grassland ecosystems (Barnard, Osborne & Firestone, 2013; Chen et al., 2017:201; Porras-Alfaro & Bayman, 2011; Tedersoo et al., 2014; Yang et al., 2017), sequences assigned to Ascomycota numerically dominated over all other fungal phyla across cultivars. The dominant fungal classes in our bahiagrass plots (Sordariomycetes, Glomeromycetes, Dothideomycetes, and Agaricomycetes) were similar to those found in Californian grassland soils (Barnard, Osborne & Firestone, 2013).

Many species of our most abundant taxonomically assigned fungal genus across cultivars, *Penicillium*, have been identified as plant growth-promoting fungi for several plants including grasses (Khan et al., 2008; Wakelin et al., 2004; Whitelaw, Harden & Bender, 1997). A well reported mechanism of plant growth promotion by *Penicillium* spp. is their ability to solubilize P for plant nutrition in soil (Asea, Kucey & Stewart, 1988; Kucey, 1987; Wakelin et al., 2004). We found that the potentially phytopathogenic genus *Fusarium* was assigned as the second most abundant genus across all plots. *Fusarium* spp. are cosmopolitans that are present in all types of ecosystems (Summerell et al., 2010) and were reported to be one of the most abundant soil fungal taxa in some grassland ecosystems (Khidir et al., 2010; Orgiazzi et al., 2012; Warcup, 1951; Yang et al., 2017). *Fusarium* diseases are, except under rare conditions, considered as not serious for bahiagrass under field conditions (Singh, 2009). Besides *Penicillium* and *Fusarium*, sequences assigned to the genus *Mortierella* were dominant and have also been shown to be highly abundant in grassland soils (Warcup, 1951; Yang et al., 2017). Members of the genus *Mortierella* are a diverse, ubiquitous and abundant group of filamentous fungi in soils that exhibit a saprophytic lifestyle (Uehling et al., 2017; Wagner et al., 2013). Additionally, some species were recently described as root endophytes (Bonito et al., 2016; Johnson et al., 2019). There is evidence that several *Mortierella* species can promote the growth of certain plant species whereby for some species, similar to *Penicillium*, one of the identified mechanisms for plant growth promotion is their ability to solubilize P for plant uptake (Osorio & Habte, 2001; Osorio & Habte, 2013; Osorio & Habte, 2014; Sharma et al., 2013; Zhang et al., 2011).

### Soil bacteria and fungi under different bahiagrass cultivars

Numerous studies have shown that plant cultivars or varieties can affect the composition of the associated soil rhizosphere bacterial and fungal communities (Bell et al., 2014; Briones et al., 2002; Dalmastri et al., 1999; Diab El Arab, Vilich & Sikora, 2001; Germida & Siciliano, 2001; Jie, Liu & Cai, 2013; Schweitzer et al., 2008). Different grass cultivars can exhibit dissimilar nutrient requirements (Ashworth et al., 2017; Oliveira et al., 2017) as well as root exudate quantities and qualities (Christiansen-Weniger, Groneman & Van Veen, 1992; Guo, McCulley & McNear, 2015), which are likely to affect populations of root-associated microorganisms. The bahiagrass cultivars differed in productivity, stand establishment and growth rate, and temperature sensitivity (Chambliss & Sollenberger, 1991; Newman, Vendramini & Blount, 2011). Thus, considering the holistic approach of plant-microbe-soil as a feedback system (Miki et al., 2010), it is likely that different bahiagrass cultivars affect the rhizosphere microbiome and alter plant-microbe-soil traits. In our study, differences in microbial community composition in response to cultivar choice were only detected for bacterial communities. It should be noted that our soil samples were a mixture of rhizosphere and bulk soil, which may have contributed to the low number of detected differences in the composition and diversity of soil microbial communities among cultivars. Soil microbial community functional diversification is thought to be crucial for soil microbiome stability and resilience (Griffiths & Philippot, 2013; Shade et al., 2012). Therefore, the comparingly low bacterial alpha diversity (Simpson’s index) and evenness (Simpson’s evenness) in TifQuik soil (Fig. 2C), may signal a decreased potential of the soil bacterial community to counter perturbations.

Differences in community composition among cultivars were limited to bacterial communities among Argentine and Sand Mountain and Argentine and TifQuik. Cultivar choice further affected relative abundance of the cosmopolitan genus *Nitrospira* (Fig. 3). *Nitrospira* affiliates are present in a wide range of habitats, including deep sea sediments (Nunoura et al., 2015), cold deserts (Gupta et al., 2015), and tropical sponges (Sharp et al., 2007). Traditionally, members of *Nitrospira* are described as nitrite-oxidizing bacteria, performing the second oxidation-step in nitrification. Recently, however, Daims et al. (2015) reported complete nitrification by a member of the genus *Nitrospira*, which completely changes our understanding of ammonia-oxidizing and nitrite-oxidizing bacteria. Apart from their place in the nitrification pathway, the increased relative abundance of *Nitrospira* under Sand Mountain compared to UF-Riata (Fig. 3) may indicate a greater potential for nitrite oxidation activity in soil of Sand Mountain. In 2014 and 2015, Dubeux et al. (2017) determined the bahiagrass yield and crude protein content of all six bahiagrass cultivars at our experimental site. The yield of Sand Mountain was among the greatest of all six cultivars and out-yielded Argentine. Although no statistically significant differences in crude protein content were detected among cultivars, it is worth mentioning that Sand Mountain showed the greatest mean crude protein content (Dubeux et al., 2017).

Wedin & Tilman (1990) reported a close relationship between soil-N cycling and the choice of perennial grass species. Several studies showed that certain grass species can suppress nitrification (Ishikawa et al., 2003; Lata et al., 2004; O’Sullivan et al., 2016; Subbarao et al., 2009). In contrast, Hawkes et al. (2005) demonstrated that invasive grass species can increase nitrification rates and the abundance of ammonia-oxidizing bacteria in soil of Californian grassland. Studies that explored the role of grass root exudates on nitrification mainly focused on nitrification inhibition as a strategy for reduced nitrate leaching from soil. Numerous studies reported nitrification inhibitors in root exudates of grasses (Subbarao et al., 2006; Subbarao et al., 2009; Sun et al., 2016; Zakir et al., 2008). The composition of grass root-exudates has been shown to be affect by both cultivar and fungal endophytes (Guo, McCulley & McNear, 2015). It remains unclear whether certain bahiagrass cultivars affect nitrification rates. However, we speculate that some cultivars may promote or less supress nitrifying soil microorganisms to increase N availability, particularly in the absence of N fertilization like at our experimental site.

Sand Mountain further harboured two indicator species, one OTU anchored in the genus *Pajaroellobacter* and the other in the genus *Bauldia* (Table S3). The genus *Pajaroellobacter* is not well characterized, except for *Pajaroellobacter abortibovis*, the etiologic agent of epizootic bovine abortion in cattle, which is a vector transmitted disease by the tick *Ornithodoros coriaceus* (Brooks et al., 2016; King et al., 2005). Likewise, the genus *Bauldia* is largely unexplored.

Sequences assigned to the genus *Haliangium* was found characteristic for the cultivars Pensacola and Tifton 9 (Table S3). *Haliangium* spp. have been recovered from soil samples before, even with great geographic distance among samples (Ding et al., 2014; Fulthorpe et al., 2008). Some members of *Haliangium* have the capability to produce the antifungal metabolite haliangicin which can supress the growth of a broad range of fungi (Fudou, Iizuka & Yamanaka, 2001; Kundim et al., 2003). There is no application of *Haliangium* in plant protection yet, however, the potential of myxobacteria to produce unique secondary metabolites has been recognized (Reichenbach & Höfle, 1993; Wenzel & Müller, 2009).

For all cultivars but Argentine, an OTU of the abundant bacterial family Nitrosomonadaceae was assigned as an indicator species (Table S1). They are characterized as lithoautotrophic of ammonia-oxidizing bacteria and harbour the well-characterized genera *Nitrosomonas* and *Nitrosospira*. In view if this result and the relative abundances of *Nitrospira*, we suggest that the dynamics of soil-N cycling under different bahiagrass cultivars should be further investigated.

Half of the cultivars (Pensacola, Sand Mountain, and Tifton 9) harboured a sequence assigned to a member of the Ceratobasidiaceae as an indicator species (Table S4). Genera of this fungal family include economically relevant phytopathogens like *Rhizoctonia*, which cause, for example, ‘brown patch’ disease on turfgrasses (Oniki et al., 1986). In rotation systems, bahiagrass has shown to reduce *Rhizoctonia* population densities in soil and associated diseases on peanuts (Johnson et al., 1999), and vegetables (cucumber (*Cucumis sativus* ‘Comet’) and snap bean (*Phaseolus vulgaris* ‘Strike’)) (Sumner et al., 1999). The two tested bahiagrass cultivars in the above-mentioned studies on peanuts and vegetables were Pensacola and Tifton 9, respectively. Since Pensacola, Sand Mountain, and Tifton 9 were characterized by an OTU assigned to a member of the Ceratobasidiaceae, our bahiagrass cultivars may differ in their ability to suppress *Rhizoctonia* population in soils. Therefore, it may be valuable to screen bahiagrass cultivars for disease suppression when used in sod-based crop rotations (i.e., 1 to 8 years of peanuts or vegetables rotated with 2 to 10 years of bahiagrass).

An OTU assigned to the widespread family Orbiliaceae was identified as an indicator species of the cultivars Sand Mountain, TifQuik, Tifton 9, and UF-Riata (Table S4). Several members of this family are carnivorous fungi which trap nematodes in soils (Pfister, 1997; Rubner, 1996). The underlying mechanisms of biocontrol of nematodes by microorganisms are well described (Li et al., 2015). Rotations of bahiagrass with peanuts, soybean (*Glycine max*), or vegetables have shown the potential to increase nematode control (Rodriguez-Kabana et al., 1988; Rodriguez-Kabana et al., 1989; Sumner et al., 1999). However, there is a lack of studies comparing the performance of different bahiagrass cultivars on nematode control. Based on our molecular results, we speculate that bahiagrass cultivar screening may improve nematode biocontrol.

## Conclusions

We detected a few differences in community composition and diversity of soil bacteria among bahiagrass cultivars, suggesting a moderate impact of cultivar choice on the soil bacterial community. Further, cultivar choice affected the relative abundance of sequences assigned to members of the nitrite-oxidizing bacterial genus *Nitrospira* with possible implications for soil-N dynamics. In contrast, soil fungal composition and diversity was not altered by the different cultivars. Several bacterial and fungal indicator species assigned to either a single cultivar or a combination of cultivars were presumptive plant pathogens or antagonists. In view of this, we suggest future work that explores the potential of bahiagrass cultivars to control plant pathogens.

##  Supplemental Information

10.7717/peerj.7014/supp-1 Figure S1 Relationship between sampling depth and the observed number of operational taxonomic units (OTUs) (A) soil bacterial and (B) fungal OTUs in plots of six different bahiagrass (*Paspalum notatum* Flüggé) cultivars (*n* = 12 for each cultivar) in a Rhodic Kandiudults soil in Northwest Florida, USA.

10.7717/peerj.7014/supp-2 Table S1 Soil pH, Mehlich-1 P, K, Ca, and Mg, and CEC of bahiagrass (*Paspalum notatum* Flüggé) plots (means ± standard error, *n* = 4) in a Rhodic Kandiudults soil in Northwest Florida, USA a, calculated cation exchange capacity.

10.7717/peerj.7014/supp-3 Table S2 PERMANOVA and PERMDSIP results for the soil bacterial and fungal community composition among six different bahiagrass (*Paspalum notatum* Flüggé) cultivars (*n* = 12 for each cultivar) in a Rhodic Kandiudults soil in Northwest Florida, USA Tests were performed based on Bray-Curtis dissimilarity matrices using 9,999 permutations. Statistically significant differences at corrected *p*-values of *p* < 0.05 are labelled with an asterisk.

10.7717/peerj.7014/supp-4 Table S3 Soil bacterial indicator species of six different bahiagrass (*Paspalum notatum* Flüggé) cultivars (*n* = 12 for each cultivar) in a Rhodic Kandiudults soil in Northwest Florida, USA Indicator species were determined using the ‘indicspecies’ R package with *α* = 0.05 and 999 permutations. Dark grey bars indicate the combination of cultivars harbouring indicator species . a, indicator value obtained from the ‘indicspecies’ R package. *p < 0.05; **p < 0.01.

10.7717/peerj.7014/supp-5 Table S4 Soil fungal indicator species of six different bahiagrass (*Paspalum notatum* Flüggé) cultivars (*n* = 12 for each cultivar) in a Rhodic Kandiudults soil in Northwest Florida, USA Indicator species were determined using the ‘indicspecies’ R package with *α* = 0.05 and 999 permutations. Dark grey bars indicate the combination of cultivars harbouring indicator species. a, indicator value obtained from the ‘indicspecies’ R package. *p < 0.05.

## Additional Information and Declarations

### Data Availability

The following information was supplied regarding data availability: Amplicon sequencing data are available at NCBI Short Read Archive (SRP143584).
https://www.ncbi.nlm.nih.gov/Traces/study/?acc=PRJNA454081
