# Supplementary figures and images for: Soil bacterial and fungal communities of six bahiagrass cultivars
Source: PeerJ. 2019 May 29;7:e7014. doi: 10.7717/peerj.7014 (PMC6545100; doi:10.7717/peerj.7014)

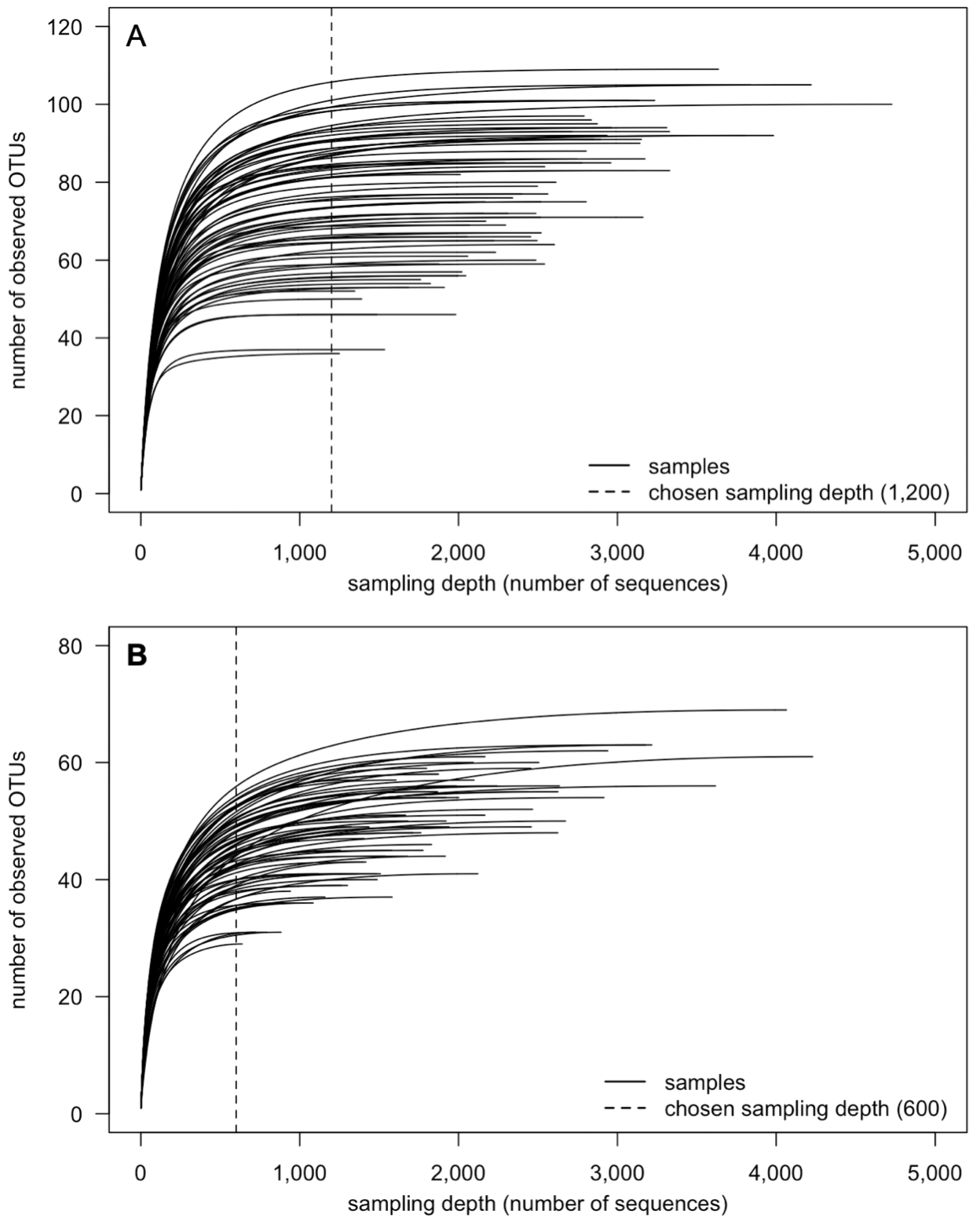

Supplement: Figure S1 — (A) soil bacterial and (B) fungal OTUs in plots of six different bahiagrass (Paspalum notatum Flüggé) cultivars (n = 12 for each cultivar) in a Rhodic Kandiudults soil in Northwest Florida, USA. [file peerj-07-7014-s001.png]
