# Supplementary material for: Soil bacterial and fungal communities of six bahiagrass cultivars
Source: PeerJ. 2019 May 29;7:e7014. doi: 10.7717/peerj.7014 (PMC6545100; doi:10.7717/peerj.7014)
Supplement: Table S1 — a, calculated cation exchange capacity. [file peerj-07-7014-s002.docx]

| Soil pH | P (kg ha^-1^) | K (kg ha^-1^) | Ca (kg ha^-1^) | Mg (kg ha^-1^) | CEC^a^  (meq 100 g^-1^) |
| --- | --- | --- | --- | --- | --- |
| 5.88  ±0.04 | 42.59  ±1.99 | 117.41  ±3.76 | 476.08  ±42.62 | 131.70  ±14.49 | 2.90  ±0.20 |
