# Supplementary material for: Soil bacterial and fungal communities of six bahiagrass cultivars
Source: PeerJ. 2019 May 29;7:e7014. doi: 10.7717/peerj.7014 (PMC6545100; doi:10.7717/peerj.7014)
Supplement: Table S2 — Tests were performed based on Bray-Curtis dissimilarity matrices using 9,999 permutations. Statistically significant differences at corrected p-values of p < 0.05 are labelled with an asterisk. [file peerj-07-7014-s003.docx]

| PERMANOVA results for soil bacterial communities | | | | | |
| --- | --- | --- | --- | --- | --- |
| compared cultivars | | | pseudo F | non-corrected *p*-value | corrected *p*-value |
| Argentine | - | Pensacola | 0.911 | 0.608 | 0.701 |
| Argentine | - | UF-Riata | 1.135 | 0.284 | 0.388 |
| Argentine | - | Sand Mountain | 1.876 | 0.003 | 0.022 |
| Argentine | - | Tifquik | 2.046 | 0.003 | 0.022 |
| Argentine | - | Tifton 9 | 1.329 | 0.116 | 0.239 |
| Pensacola | - | UF-Riata | 0.961 | 0.523 | 0.654 |
| Pensacola | - | Sand Mountain | 1.535 | 0.043 | 0.107 |
| Pensacola | - | Tifquik | 1.820 | 0.014 | 0.068 |
| Pensacola | - | Tifton 9 | 1.307 | 0.128 | 0.239 |
| UF-Riata | - | Sand Mountain | 1.523 | 0.034 | 0.102 |
| UF-Riata | - | Tifquik | 1.626 | 0.034 | 0.102 |
| UF-Riata | - | Tifton 9 | 0.671 | 0.936 | 0.936 |
| Sand Mountain | - | Tifquik | 1.248 | 0.193 | 0.321 |
| Sand Mountain | - | Tifton 9 | 1.166 | 0.248 | 0.373 |
| Tifquik | - | Tifton 9 | 0.841 | 0.689 | 0.738 |
|  | | | | | |
| PERMDISP results for soil bacterial communities | | | | | |
|  | | | | | |
| compared cultivars | | | pseudo F | non-corrected *p*-value | corrected *p*-value |
| Argentine | - | Pensacola | 0.229 | 0.620 | 0.875 |
| Argentine | - | UF-Riata | 0.537 | 0.467 | 0.875 |
| Argentine | - | Sand Mountain | 0.201 | 0.641 | 0.875 |
| Argentine | - | Tifquik | 0.005 | 0.947 | 0.954 |
| Argentine | - | Tifton 9 | 0.948 | 0.311 | 0.875 |
| Pensacola | - | UF-Riata | 0.117 | 0.734 | 0.918 |
| Pensacola | - | Sand Mountain | 0.004 | 0.954 | 0.954 |
| Pensacola | - | Tifquik | 0.315 | 0.543 | 0.875 |
| Pensacola | - | Tifton 9 | 2.273 | 0.129 | 0.875 |
| UF-Riata | - | Sand Mountain | 0.056 | 0.824 | 0.950 |
| UF-Riata | - | Tifquik | 0.642 | 0.426 | 0.875 |
| UF-Riata | - | Tifton 9 | 2.606 | 0.116 | 0.875 |
| Sand Mountain | - | Tifquik | 0.268 | 0.576 | 0.875 |
| Sand Mountain | - | Tifton 9 | 1.691 | 0.194 | 0.875 |
| Tifquik | - | Tifton 9 | 0.865 | 0.346 | 0.875 |
|  | | | | | |
| PERMANOVA results for soil fungal communities | | | | | |
| compared cultivars | | | pseudo F | non-corrected *p*-value | corrected *p*-value |
| Argentine | - | Pensacola | 1.153 | 0.290 | 0.486 |
| Argentine | - | UF-Riata | 1.108 | 0.331 | 0.486 |
| Argentine | - | Sand Mountain | 0.693 | 0.813 | 0.871 |
| Argentine | - | Tifquik | 1.278 | 0.194 | 0.486 |
| Argentine | - | Tifton 9 | 1.454 | 0.089 | 0.486 |
| Pensacola | - | UF-Riata | 0.537 | 0.969 | 0.969 |
| Pensacola | - | Sand Mountain | 1.303 | 0.163 | 0.486 |
| Pensacola | - | Tifquik | 1.492 | 0.071 | 0.486 |
| Pensacola | - | Tifton 9 | 1.063 | 0.358 | 0.486 |
| UF-Riata | - | Sand Mountain | 1.330 | 0.148 | 0.486 |
| UF-Riata | - | Tifquik | 1.190 | 0.247 | 0.486 |
| UF-Riata | - | Tifton 9 | 1.033 | 0.414 | 0.486 |
| Sand Mountain | - | Tifquik | 1.032 | 0.403 | 0.486 |
| Sand Mountain | - | Tifton 9 | 1.149 | 0.284 | 0.486 |
| Tifquik | - | Tifton 9 | 1.027 | 0.421 | 0.486 |
|  | | | | | |
| PERMDISP results for soil fungal communities | | | | | |
|  | | | | | |
| compared cultivars | | | pseudo F | non-corrected *p*-value | corrected *p*-value |
| Argentine | - | Pensacola | 0.089 | 0.756 | 0.835 |
| Argentine | - | UF-Riata | 0.518 | 0.440 | 0.835 |
| Argentine | - | Sand Mountain | 1.361 | 0.186 | 0.835 |
| Argentine | - | Tifquik | 0.301 | 0.551 | 0.835 |
| Argentine | - | Tifton 9 | 1.631 | 0.173 | 0.835 |
| Pensacola | - | UF-Riata | 0.604 | 0.418 | 0.835 |
| Pensacola | - | Sand Mountain | 1.058 | 0.281 | 0.835 |
| Pensacola | - | Tifquik | 0.420 | 0.491 | 0.835 |
| Pensacola | - | Tifton 9 | 1.286 | 0.247 | 0.835 |
| UF-Riata | - | Sand Mountain | 0.115 | 0.706 | 0.835 |
| UF-Riata | - | Tifquik | 0.014 | 0.902 | 0.902 |
| UF-Riata | - | Tifton 9 | 0.203 | 0.626 | 0.835 |
| Sand Mountain | - | Tifquik | 0.160 | 0.662 | 0.835 |
| Sand Mountain | - | Tifton 9 | 0.063 | 0.779 | 0.835 |
| Tifquik | - | Tifton 9 | 0.312 | 0.553 | 0.835 |
