# Supplementary material for: Soil bacterial and fungal communities of six bahiagrass cultivars
Source: PeerJ. 2019 May 29;7:e7014. doi: 10.7717/peerj.7014 (PMC6545100; doi:10.7717/peerj.7014)
Supplement: Table S3 — Indicator species were determined using the ‘indicspecies’ R package with α = 0.05 and 999 permutations. Dark grey bars indicate the combination of cultivars harbouring indicator species . a, indicator value obtained from the ‘indicspecies’ R package. *p < 0.05; **p < 0.01. [file peerj-07-7014-s004.docx]

| Argentine | Pensacola | Sand Mountain | TifQuik | Tifton 9 | UF-Riata | Indicator species (phylum - order - family - genus - species) | Indicator  value^a^ | *p*-value |
| --- | --- | --- | --- | --- | --- | --- | --- | --- |
|  |  |  |  |  |  |  |  |  |
|  |  |  |  |  |  | Proteobacteria - Myxococcales - Polyangiaceae - *Pajaroellobacter* - unassigned sp. | 0.493 | 0.009** |
|  |  |  |  |  |  | Proteobacteria - Alphaproteobacteria - Rhizobiales - Rhizobiales Incertae Sedis - *Bauldia* - unassigned sp. | 0.489 | 0.048* |
|  |  |  |  |  |  |  |  |  |
|  |  |  |  |  |  | Chloroflexi - RBG-13-54-9 - unassigned family - unassigned genus - unassigned sp. | 0.483 | 0.019* |
|  |  |  |  |  |  |  |  |  |
|  |  |  |  |  |  | Proteobacteria - Rickettsiales - SM2D12 - unassigned genus - unassigned sp. | 0.437 | 0.048* |
|  |  |  |  |  |  |  |  |  |
|  |  |  |  |  |  | Fibrobacteres - Fibrobacterales - Fibrobacteraceae - unassigned genus - unassigned sp. | 0.557 | 0.007** |
|  |  |  |  |  |  |  |  |  |
|  |  |  |  |  |  | Actinobacteria - Microtrichales - unassigned family - unassigned genus - unassigned sp. | 0.540 | 0.008** |
|  |  |  |  |  |  | Proteobacteria - Myxococcales - Haliangiaceae - *Haliangium* - unassigned sp. | 0.475 | 0.024* |
|  |  |  |  |  |  |  |  |  |
|  |  |  |  |  |  | Bacteroidetes - Cytophagales - Microscillaceae - unassigned genus - unassigned sp. | 0.540 | 0.036* |
|  |  |  |  |  |  |  |  |  |
|  |  |  |  |  |  | Proteobacteria - Rhizobiales - Methyloligellaceae - unassigned genus - unassigned sp. | 0.616 | 0.016* |
|  |  |  |  |  |  |  |  |  |
|  |  |  |  |  |  | Acidobacteria - Subgroup 2 - unassigned family - unassigned genus - unassigned sp. | 0.948 | 0.023* |
|  |  |  |  |  |  | Proteobacteria - Myxococcales - bacteriap25 - unassigned genus - unassigned sp. | 0.792 | 0.036* |
|  |  |  |  |  |  | Chloroflexi - unassigned order - unassigned family - unassigned genus - unassigned sp. | 0.765 | 0.038* |
|  |  |  |  |  |  |  |  |  |
|  |  |  |  |  |  | Proteobacteria - Betaproteobacteriales - Nitrosomonadaceae - mle1-7 - unassigned sp. | 0.803 | 0.017* |
|  |  |  |  |  |  | Proteobacteria - Betaproteobacteriales - SC-I-84 - unassigned genus - unassigned sp. | 0.743 | 0.009** |
