# Supplementary material for: Soil bacterial and fungal communities of six bahiagrass cultivars
Source: PeerJ. 2019 May 29;7:e7014. doi: 10.7717/peerj.7014 (PMC6545100; doi:10.7717/peerj.7014)
Supplement: Table S4 — Indicator species were determined using the ‘indicspecies’ R package with α = 0.05 and 999 permutations. Dark grey bars indicate the combination of cultivars harbouring indicator species. a, indicator value obtained from the ‘indicspecies’ R package. *p < 0.05. [file peerj-07-7014-s005.docx]

| Argentine | Pensacola | Sand Mountain | TifQuik | Tifton 9 | UF-Riata | Indicator species (phylum - order - family - genus - species) | Indicator  value^a^ | *p*-value |
| --- | --- | --- | --- | --- | --- | --- | --- | --- |
|  |  |  |  |  |  |  |  |  |
|  |  |  |  |  |  | Ascomycota - Helotiales - Helotiaceae - unassigned genus - unassigned sp. | 0.500 | 0.024* |
|  |  |  |  |  |  |  |  |  |
|  |  |  |  |  |  | Chytridiomycota - Spizellomycetales – Spizello-mycetaceae - unassigned genus - unassigned sp. | 0.500 | 0.030* |
|  |  |  |  |  |  |  |  |  |
|  |  |  |  |  |  | Ascomycota - Pezizales - Pezizaceae - unassigned genus - unassigned sp. | 0.697 | 0.029* |
|  |  |  |  |  |  |  |  |  |
|  |  |  |  |  |  | Ascomycota - Orbiliales - Orbiliaceae - unassigned genus - unassigned sp. | 0.536 | 0.030* |
|  |  |  |  |  |  |  |  |  |
|  |  |  |  |  |  | Basidiomycota - Cantharellales - Ceratobasidiaceae - unassigned genus - unassigned sp. | 0.698 | 0.016* |
|  |  |  |  |  |  |  |  |  |
|  |  |  |  |  |  | Ascomycota - Coniochaetales - Coniochaetaceae - unassigned genus - unassigned sp. | 0.637 | 0.040* |
